# Impact of (recurrent) bacterial vaginosis on quality of life and the need for accessible alternative treatments

**DOI:** 10.1186/s12905-023-02236-z

**Published:** 2023-03-18

**Authors:** Karen Chow, Darcy Wooten, Sindhuja Annepally, Leah Burke, Rina Edi, Sheldon R. Morris

**Affiliations:** Department of Medicine, University of California San Diego, San Diego, CA USA

**Keywords:** Bacterial vaginosis, Recurrent bacterial vaginosis, Boric acid, Lactobacillus, Alternative therapies

## Abstract

**Background:**

Bacterial vaginosis (BV) is one of the most common vaginal dysbiosis in women aged 15–44 years old.

**Methods:**

We administered a cross-sectional, single timepoint survey to women ages 18 years or older and who have had bacterial vaginosis (BV). Women completed an anonymous online survey evaluating the impact of BV on their quality of life, how effective different types of treatments were and the amount of self-diagnosed vs. provider diagnosed BV episodes they had.

**Results:**

62 participants completed the anonymous online survey. With a self-reported median number of BV episodes in the past year was 4 (IQR 1–7). Among these women 69.8% reported BV had a negative impact on their sexual health, 67.7% on their physical health, 74.6% on their mental health. More than half of the respondents had used probiotics with oral Lactobacillus sp. (53.2%), mainly by oral route, and over a third had used vaginal boric acid (37.1%). Most women were unaware of *Lactobacillus crispatus*. Lactobacillus probiotics were more likely to be tried by women who were negatively impacted by BV for overall quality of life (*p* = 0.033), sexual health (*p* = 0.002), and mental health (*p* = 0.006) while boric acid use was more likely to be used by women who were negatively impacted by BV for their sexual health (*p* = 0.008).

**Conclusions:**

BV is associated with negative quality of life and the women most impacted are seeking alternative treatments such as probiotics (Lactobacillus) and boric acid. There needs to be improvements in BV treatment that include alternative therapy options that have demonstrated efficacy with standardized composition, formulation and dosage.

## Introduction 

The Centers of Disease Control and Prevention (CDC) reports that bacterial vaginosis (BV) is the most common vaginal condition in women aged 15–44 years old [1]. It is very common for BV to recur despite successful antibiotic treatment; yet there is limited management strategies for recurrent disease [2]. Studies have shown that women are dissatisfied with taking frequent and repeated courses of antibiotics, and many turn to self-help remedies and lifestyle modifications [3]. Additionally, when experiencing frequent episodes it is inconvenient to seek repeated diagnostic testing for vaginitis for which may involve a vaginal exam by a qualified healthcare provider and evaluation of the Amsel criteria (vaginal pH greater than 4.5, positive whiff test, presence of clue cells on microscope, and abnormal vaginal discharge) [4]. Treatment of BV is important because it may increase the risk for: sexually transmitted infections (e.g., chlamydia, gonorrhea, and HIV), pelvic inflammatory disease, infertility, and preterm labor [5–7]. The current CDC-recommended regimen for BV is 500 mg of oral metronidazole twice a day for 7 days. [2] Because of the side effects of metronidazole, such as nausea, vomiting, other gastrointestinal complaints, and/or concern about overexposure to antibiotics, some women do not find repeated course of metronidazole a sustainable long-term strategy for recurrent BV.

Given the high number of women who suffer from recurrent BV, enhancing and developing new treatment options that are effective is a high priority. Previous studies have shown that women have BV recurrence rates of up to 69% within 12 months of the CDC recommended current treatments [8, 9]. High rates of recurrences underscore that current therapies such as oral metronidazole, intravaginal metronidazole or intravaginal clindamycin do not directly restore the normal vaginal microflora [10]. Recent studies have suggested that probiotics may play a role in the treatment of BV when combined with antibiotics [10, 11]. Alternative approaches to therapy such as *Lactobacillus crispatus* (LACTIN-V) vaginal inserts have shown some success in preventing recurrence of BV [12]. One of the major barriers to effective treatment is vaginal biofilms which limit antibiotic penetration and thus antimicrobial efficacy. Biofilm production is a hallmark feature of *Gardnerella vaginalis*, one of the dominant species associated with recurrent BV [13]. Biofilm-related infections tend to persist since antibiotics are unable to fully eradicate them. Previous studies have shown that lactobacilli can both block the ability of pathogens to form a biofilm and produce antimicrobial substances such as hydrogen peroxide to eradicate pathogens [14]. Alternative strategies such as intravaginal boric acid which alters microflora through acidification of the vagina, have also shown promise but require more randomized controlled studies to confirm its efficacy [15, 16]. In one study which utilized a combination of oral nitroimidazole and topical boric acid, there was high success rates after only 30 days and treatment was well tolerated [17]. To further understand how frequently women with BV are trying these alternative therapies and what factors might be associated with alternative therapies, this study surveyed women who had previously had a diagnosis of BV on frequency of BV, impact of BV on their lives and what treatments they have tried.

## Methods

We administered an online survey between September 2021 and March 2022 to English-speaking participants who have had recurrent BV. We obtained Investigational Review Board approval from UC San Diego (HRPP# 191,376) and online informed consent prior to administration and completion of this anonymous online survey. The survey objective was to assess the impact of BV on quality of life and what BV treatments they have tried to determine what factors might be associated with use of alternative therapies. Participants were recruited through multiple methods: through previous participation in the LACTIN-V (NCT02766023) and BV SUBVert (NCT03930745) studies, by provider referral from investigators, via the website ResearchMatch, via advertising from StudyKik, and by participant referrals. The eligibility criteria included: being assigned female sex at birth, being 18 years or older, being English-speaking, and having had BV anytime in the past. Survey data were collected and managed using REDCap electronic data capture tools hosted at UC San Diego [18].

The survey instrument comprised of 24 items that collected: demographics (age, race/ethnicity, income, education, and employment status), types of sexual partnerships, frequency of BV episodes, impact of BV on quality of life, types of treatments tried, and preferences for treatments. Survey questions were composed to evaluate number of recurrences, which types of treatments had been tried, and how BV had impacted specific components to their quality of life. Responses utilized a five-point Likert scale (e.g., very positively to very negatively). Quality of questions were reviewed by study team members prior to publishing the final instrument.

To determine the frequency of BV symptoms and episodes, the survey asked participants to answer with a whole number: “in the past year, how many times would you say you have had symptoms of BV” and “in the past year, how many times did you get the diagnosis of BV by a medical provider?” Using a five-point Likert scale, we assessed the impact of BV on quality of life across the following domains: interactions with others, sexual relationships, mental health, physical health, and overall quality of life. Levels of response included ‘very positive’, ‘positive’, ‘neutral’, ‘negative’ and ‘very negative’. To evaluate the importance of BV as a health concern, the survey asked participants to answer using a four-point Likert scale: “how important of a health issue to you is your BV?” To assess the efficacy of available treatment options, the survey asked participants to select all the types of treatments they have tried: oral metronidazole, oral tinidazole, metronidazole gel, probiotics, boric acid, and other. Survey data did not include any patient identification and participants could decline to answer certain questions.

All analyses were conducted using SPSS 27.0 statistical software package [19]. Descriptive statistics was used to calculate the frequencies and percentages for demographic data. Cross-tabs and the Fisher exact test were used for determining relationships between categorical variables of products used and impact on health outcomes that were dichotomized as “negative/very negative’ compared to ‘neutral/positive/very positive’. Significance was determined as a two-sided *p* ≤ 05. 

## Results

There were 62 respondents to the survey, two thirds of respondents were ages 31–50 with 46.7% white, 22.5% Latina, 14.5% Black and 16.3% other race/ethnicity (Table 1). Most of the respondents were menstruating in the past year (80.6%) and only 32.3% were on hormonal therapy for contraception or other reason.

**Table 1:** Demographic characteristics of survey participants

	Frequency (*n* = 62)	Percent (%)
*Age Range (years)*		
18—25	6	9.7
26—30	13	21.0
31 – 50	41	66.1
> 50	2	3.2
*Race*		
Asian	3	4.8
Hispanic or Latino	14	22.5
White	29	46.7
Black/African American	9	14.5
Native American	2	3.2
Mixed	5	8.1
Prefer not to answer	1	1.6
*Highest Education Level*		
Bachelor’s Degree	22	35.5
High School Diploma or GED	19	30.6
Less than High School Diploma	0	0.0
Do not wish to report	1	1.6
Advanced Degree (Master’s, Ph.D., MD)	20	32.3
*Employment*		
Yes	48	77.4
No	14	22.6
*Income (Annual)*		
$1–9,999	3	4.8
$10,000–49,999	19	30.6
$50,000–74,999	7	11.3
$75,000–99,999	10	16.1
$100,000–149,999	7	11.3
> $150,000	9	14.5
Do not wish to report	7	11.3

The median number of self-diagnosed BV episodes in the past year was 4 (IQR 1–7). The most common symptoms that women experienced were vaginal odor (83.9%), vaginal discharge (82.3%), vaginal irritation/itchiness (67.7%), pain with intercourse (27.4%), spotting between menses (16.1%) and cramping (12.9%). The median number of provider-diagnosed BV episodes in the past year was 2 (IQR 0–4). Most participants were diagnosed by a Family Physician (33.9%) or a Gynecologist (32.3%) and a small proportion were diagnosed at a Family Planning Clinic (17.7%). Forty percent of respondents reported also having one or more non-BV genital tract infection in the past year. Of these, 80% had vaginal candidiasis, 12% trichomonas, 4% herpes, 16% HPV and 4% gonorrhea.

Respondents reported receiving a variety of antimicrobial treatments for BV in the past year including oral metronidazole (77.4%), metronidazole vaginal gel (69.4%) and oral tinidazole (16.1%). More than half of the respondents had used probiotics with oral Lactobacillus sp. (53.2%) and over a third had used vaginal boric acid (37.1%). Of those who tried probiotics most used oral formulations (*n* = 28) with a fewer reports of intravaginal administration (*n* = 9). Only 15 (23.8%) women had heard of *Lactobacillus crispatus*. When asked the preferred route for treatment women were split between oral (45.2%) and vaginal (54.8%). Eight (12.9%) participants reported using a variety of alternative products for BV treatment including folic acid, cranberry juice, raspberry leaf tea, intravaginal probiotics, menstrual cup, cloth pads, ozone, Vagisil, dilute hydrogen peroxide douche, garlic, yogurt, tea tree oil, vinegar, coconut oil, castor oil, oregano, kefir, ACV spray and rephresh gel.

Of 62 participants that responded, 66.1% reported that BV negatively impacted their overall quality of life, 71.0% reported a negative impact on their sexual health, 54.8% on their physical health, 75.8% on their mental health, and 29.0% on their social interactions with other people. Women who were negatively impacted by BV for overall quality of life (*p* = 0.033), sexual health (*p* = 0.002), and mental health (*p* = 0.006), were all more likely to have tried Lactobacillus probiotics than those who did not report negative impact from their BV (Table 2). Boric acid was more likely to be used by women who were negatively impacted by BV for sexual health (*p* = 0.009). In those negatively impacted by BV using Boric Acid for treatment, no significant difference was reported in physical health, mental health and social interactions.

**Table 2:** Effects of Bacterial vaginosis on Health by Use of Alternative Treatments

	BV has Negative/ Very Negative Impact	Has used lactobacillus probiotic			Has used boric acid		
		Yes N (Col. %) 33 (100%)	No N (Col. %) 29 (100%)	p	Yes N (Col. %) 23 (100%)	No N (Col. %) 39 (100%)	p
Quality of life	Yes No	26 (86.7%) 7 (13.3%)	15 (51.2%) 14 (48.8%)	0.033	19 (82.6%) 4 (17.4%)	22 (56.4%) 17 (43.6%)	0.052
Sexual health	Yes No	29 (87.9%) 4 (12.1%)	15 (51.2%) 14 (48.8%)	0.002	21 (91.3%) 2 (8.7%)	23 (59.0%) 16 (41.0%)	0.009
Physical health	Yes No	22 (66.7%) 11 (33.3%)	12 (41.4%) 17 (58.6%)	0.073	13 (56.5%) 10 (43.5%)	21 (53.8%) 18 (46.2%)	1.000
Mental health	Yes No	30 (90.9%) 3 (9.1%)	17 (58.6%) 12 (41.4%)	0.006	20 (87.0%) 3 (13.0%)	27 (93.1%) 12 (6.9%)	0.138
Social interactions	Yes No	10 (30.3%) 23 (69.7%)	8 (27.6%) 21 (82.4%)	1.000	7 (30.4%) 16 (69.6%)	11 (37.9%) 28 (42.1%)	1.000

## Discussion

Despite the potential health implications, current available treatment options for (recurrent) BV is limited and ineffective [2]. Even with successful antibiotic treatment, reported rates of relapses are frequent, with more than 50% relapsing within 3–6 months [20]. Using metronidazole as the first line of treatment as a monotherapy may be insufficient for treatment of recurrent BV as its restoration of healthy vaginal flora is likely only temporary. In a previous study that analyzed the microbiome of 63 women (17 patients with recurrent BV compared to 46 healthy controls), lactobacilli was less abundant in women with recurrent BV [21]. After one week of oral metronidazole treatment, their vaginal microbiomes had an increase in lactobacilli. However, these changes were not sustained one month after treatment. Due to this persistent vaginal dysbiosis, there recurrence of BV symptoms is very problematic for many women. A recent network meta-analysis reviewed clinical cure rates (CCR) of various BV therapies in randomized control trials across the globe. Despite significant heterogenicity in CCR’s, the highest CCR’s were calculated using combined antibiotic and probiotic therapy, with the lowest CCR’s noted in non-combined therapies [10]. With previous studies focused on women’s experiences with BV, most women reported it had a moderate to severe effect physically, emotionally, sexually, and socially; the level of impact was dependent on the frequency of episodes and severity of symptoms [22]. Our results found similarly that women with recurrent BV reported negative impact in multiple domains. Our survey found the women who were impacted negatively by recurrent BV also were more likely to try alternative therapies, many of which are not FDA-regulated nor are they tested for efficacy or safety. The most common alternative therapy used by participants in our study was probiotics. A negative impact on overall quality of life, sexual health or mental health was associated with the use of Lactobacillus probiotics. Most probiotic use was oral and less than a quarter were aware of the key probiotic microorganism (*Lactobacillus Crispatus)*. Use of vaginal boric acid was most strongly associated with women reporting negative effects of BV on sexual health.

Limitations of this study include the relatively small sample size (*n* = 62) derived mostly from only one geographic location (San Diego). The majority of participants were also recruited from previous clinical trials with boric acid and Lactobacillus, which may have introduced a bias towards alternative therapies. Most participants had completed college or higher education and therefore may represent a more informed cohort. The survey did not use longer validated instruments to measure quality of life to minimize the effort needed to complete the survey. Actual number of BV recurrences may be inaccurate as the self-reported cases may not actually be BV and those that were diagnosed by a provider may not include all the cases of BV that a woman experienced.

In conclusion, our finding suggests women who are highly impacted by BV are more likely to try alternative treatments such as boric acid and Lactobacillus probiotics but also a multitude of other products. The results indicate that standardized commercial probiotic and boric acid products need to be developed with definitive clinical studies. Education of primary care providers may help to counsel women on BV etiology and how they might use possible alternative approaches.

## Data Availability

The datasets generated and/or analyzed during the current study are not publicly available due to privacy restrictions but are available from the corresponding author on reasonable request.
